# Rhythmic Auditory Stimulation as an Adjuvant Therapy Improved Post-stroke Motor Functions of the Upper Extremity: A Randomized Controlled Pilot Study

**DOI:** 10.3389/fnins.2020.00649

**Published:** 2020-06-30

**Authors:** Rujin Tian, Bei Zhang, Yulian Zhu

**Affiliations:** ^1^Department of Rehabilitation Medicine, Huashan Hospital, Fudan University, Shanghai, China; ^2^Sports Medicine and Rehabilitation Center, Qingdao Municipal Hospital, Qingdao, China; ^3^Department of Physical Medicine and Rehabilitation, McGovern Medical School, The University of Texas Health Science Center at Houston, Houston, TX, United States

**Keywords:** rhythmic auditory stimulation, stroke, motor function, upper extremity, surface EMG

## Abstract

**Objectives:**

To explore whether rhythmic auditory stimulation (RAS) could improve motor functions of post-stroke hemiparetic upper extremity.

**Design:**

A prospective, randomized controlled, assessor-blinded pilot study.

**Methods:**

Thirty stroke patients were randomly distributed into the RAS group (*n* = 15) and the control group (*n* = 15). Both groups received regular therapies. The RAS group received additional 30 min of RAS training, while the control group received additional 30 min of regular therapies for 5 days per week for 4 weeks. The Fugl-Meyer Assessment—Upper Extremity (FMA-UE), Wolf Motor Function Test (WMFT), and Barthel Index (BI) were used. The co-activation interval and co-contraction index were calculated from surface electromyography (sEMG) recordings on the affected biceps and triceps during elbow flexion and extension. Assessments were performed before and after the treatments.

**Results:**

Significant improvements in motor functions were observed within both groups (*p* < 0.05 in the FMA-UE, WMFT, and BI, respectively), as well as between groups after the treatments (higher scores in the RAS group, all *p* < 0.05 except for *p* = 0.052 in the FMA-UE; group × time interaction, all *p* < 0.05). Statistical significance was found in the co-activation interval between groups after the treatments (lower in the RAS group; *p* = 0.022 during elbow extension; *p* = 0.001 during elbow flexion; group × time interaction, *p* < 0.05 only during elbow extension). No statistical significance was found in the co-contraction index between groups; an inversed pattern of changes was observed between groups supported by relatively higher increments in the triceps recruitments to the biceps.

**Conclusion:**

Using RAS in task-oriented exercises was effective in moderating co-contraction, facilitating task-oriented movements of the hemiparetic upper extremity, and improving ADLs among those who had emerging isolated joint movements. The effects were evident on sEMG possibly by adjusting the balance of recruitments between the agonist and the antagonist.

**Clinical Trial Registration:**

The study was registered at the Chinese Clinical Trial Registry (No. 1900026665).

## Introduction

Stroke commonly results in impairments on limbs’ functions (Hu et al., 2019), leaving patients with various levels of dependence (WHO, 2014; Zerna et al., 2019). Motor function of the upper extremity is one of the cardinal determinants of functional independence and quality of life (Kokotilo et al., 2009; Pollock et al., 2014). It has been a challenge to restore due to the complicated involvement of multiple motor domains (i.e., weakness, spasticity, incoordination, and synergistic movements) in the arm and hand after stroke (Padua et al., 2019), yet the intricacy of neural wiring and plasticity remains unclear. Task-oriented movement therapy was considered beneficial in the restoration of upper extremity functions (Boffa et al., 2019) as it emphasizes on movements highly relevant to daily activities, which theoretically facilitates neural plasticity toward a practical formation and has been routinely incorporated in the current rehabilitative practice. However, patients, even with substantial functional recovery, still struggle with the trajectory and the efficiency of using the affected arm. There is a clinical need for further exploration of potential therapeutic modalities to maximize motor functions of the paretic upper extremity.

The RAS, as a type of neurologic music therapy, was found to be effective in improving motor functions, especially gait, in persons with neurological disorders (Schaffert et al., 2019). Numerous studies have shown that RAS improved gait velocity, stride length, and cadence after stroke, as well as balance and sit-to-stand and walk sequencing (Thaut et al., 2007; Hayden et al., 2009; Cha et al., 2014; Suh et al., 2014). There are comparatively fewer studies focusing on its application and efficacy on upper extremity motor functions (Yoo and Kim, 2016; Ghai, 2018). Despite the scarcity of the study and the heterogeneity in the available studies, the pooled data in a meta-analysis favored RAS on post-stroke upper extremity motor functions (Yoo and Kim, 2016). The beneficial effects manifested in strength, range of motion, synchrony, coordination, and functional motor performance (Whitall et al., 2000; Malcolm et al., 2009; Ghai, 2018). The major deficiencies in these studies were small sample size and without control subjects.

The proposed theory on RAS’s therapeutic effects include the regulation of spatiotemporal and force parameters with specification of the dynamics of a movement and reduction of variability via the rhythmic cues, thus achieving the optimal movement pattern with repetitive exercise (Thaut et al., 2002; Thaut et al., 2009; Kim et al., 2014). Also, the rhythmic auditory cues may help with motor priming, facilitate movement anticipation and preparation, and potentially bypass damaged areas through the activation of alternative pathways (Yoo and Kim, 2016). Part of the auditory-motor coupling was proposed to locate in the RS pathway. It was suggested, theoretically, that this type of cues would activate RS pathway and result in synergistic activation, thus being more helpful in gross motor strength in patients with less spasticity but severe paresis, rather than those with more spasticity and spastic co-contraction (Li, 2017). In the study, the sEMG was used to further explore the changes of neuromuscular activities under the rhythmic auditory cues, as previously used in the gait analysis after RAS therapy, to provide new evidence on its neuromuscular modulation effects (Schreiber et al., 2016).

Therefore, the aim of this study was to explore the effects and mechanisms of RAS on the hemiparetic upper extremity motor functions after stroke, using clinical scales for functional evaluations and sEMG as an objective measurement of the underlying neuromuscular changes.

## Materials and Methods

### Study Design

This was a prospective, randomized controlled study. The sample size of 30 subjects was considered sufficient to manifest statistical significance based on previous study protocols (Hsieh et al., 2009; Schreiber et al., 2016) and a sample size calculation based on an effect size of 0.97, a significance level of 0.05, and 80% power. It was derived from two previous studies in which the effect sizes were 0.83 and 1.12 on the FMA, respectively (Whitall et al., 2000; van Delden et al., 2013). Considering approximately 10% dropout rate, the sample size was determined as 32 subjects. Eligible stroke patients were recruited and assigned a number orderly on a pre-established random number list in the Microsoft Excel software. The numbers on the list were rearranged in an ascending order, with the first 16 numbers on the sequence being assigned to the RAS group while the latter 16 numbers being assigned to the control group. The study was reviewed and approved by the Ethics Committee of Huashan Hospital, Fudan University. The study was registered at the Chinese Clinical Trial Registry (No. 1900026665).

### Participants

The inclusion criteria were (I) confirmed diagnosis of stroke with evidence on MRI or CT; (II) having motor impairments in the upper extremity with a Brunnstrom Stages IV–VI; (III) first-time stroke with or without previous lacunar infarction which resulted in no functional consequences; (IV) 40–80 years old; (V) vital signs stable; and (VI) inpatient rehabilitation status. The exclusion criteria were (I) having Parkinson’s disease or other neurological conditions causing motor dysfunction; (II) having cognitive (MMSE < 24) or auditory (tuning-fork test) impairment; (III) having cancer or severe cardiopulmonary diseases; (IV) participating in other research projects; (V) unable to follow commands; and (VI) having pacemaker placement. All participants signed the informed consent and participated under their free will.

### Interventions

All participants received regular therapies, including 30 min of individualized physical therapy and 30 min of individualized occupational therapy per day, 5 days per week for 4 weeks. The patients in the RAS group received additional 30 min of RAS therapy every day, while the patients in the control group received additional 15 min of regular physical therapy and 15 min of regular occupational therapy every day, which were also provided 5 days per week for 4 weeks.

#### Regular Therapies

The regular physical therapy included strength exercise (e.g., isotonic movements with weights and repetitions), gait exercise, balance exercise, and coordination exercise (e.g., side walking exercise and heel-to-toe walking exercise). The regular occupational therapy included forced use of the affected upper extremity in ADL activities, fine motor exercise of the hand (e.g., grasp a cylindrical object or a small bead and move to the target area), and sensory integration (e.g., mold with plasticine or squeeze soft ball).

#### RAS Therapy

The RAS therapy was performed by practicing movements of certain tasks with auditory cues at a gradually increased rhythm, as delineated in Table 1. Two categories of tasks were chosen. Task Numbers 1–9 without usage of instruments (Category I) were provided during the first 2 weeks; Task Numbers 10–14 with usage of certain instruments (Category II) were performed during the latter 2 weeks. The process of proceeding RAS therapy is described as below. First, to identify the applicable tempo (beats per minute, bpm) at the beginning of the study, the participants were required to perform each movement for 5 rounds at their own pace without the auditory cues. The trial was recorded and analyzed by a metronome software (Pro Metronome, 2014 EUMLab, Xanin Technology Limited Liability Company, China) to obtain the baseline tempo for each movement. For example, for the 3rd movement listed in Table 1, the participant was required to touch the target line (5 cm in width) back and forth by flexing and extending the shoulder. The metronome software recorded and calculated the average tempo of the movement, which was considered as the baseline. When the participant was able to keep up with a tempo and finish touching the target lines for 5 rounds, the tempo would be increased by 5% as the next level of training. No more than 5% increase was allowed in a day. Repetitive exercise with a specific tempo was implemented under supervision and instruction of an experienced therapist until the next level could be reached. The tempos at the beginning and at the end of the study were recorded, of which the means and the increments were calculated and listed in Table 1.

**TABLE 1:** The rhythmic auditory stimulation protocol.

**Number**	**Movement**	Specification*	**Tempo 1 (bpm)^#^**	**Tempo 2 (bpm)^§^**	**Increment of the tempo**
**Category I:**					
(1)	Shrug bilateral shoulder	Shrug and relax	40 ± 2	60 ± 3	50%
(2)	Shrug affected side	Shrug and relax	40 ± 1	60 ± 1	50%
(3)	Shoulder flexion/extension to touch the target line	With elbow extension	53 ± 4	80 ± 4	51%
(4)	Shoulder abduction/adduction to touch the target line	With elbow extension	54 ± 3	79 ± 4	46%
(5)	Elbow flexion/extension to touch the nose and the knee back and forth	Use index fingers to touch	51 ± 4	79 ± 4	55%
(6)	Reach back and forth on a desk	Elbow extension/flexion combined with shoulder flexion/extension	52 ± 4	79 ± 4	52%
(7)	Forearm pronation/supination	Palm contact desk when pronation; Opisthenar contact desk when supination;	64 ± 2	88 ± 4	38%
(8)	Wrist flexion/extension	Without compensatory action	65 ± 1	98 ± 4	51%
(9)	Shoulder horizontal abduction/adduction to touch the target line	With elbow extension (1 action per 2 beats)	69 ± 2	87 ± 4	26%
**Category II:**					
(10)	Hold a cup to move	Use it to touch mouth and put it back on the desk	52 ± 4	70 ± 4	35%
(11)	Hold a large block to move	Put the block in the target scope	64 ± 2	79 ± 2	23%
(12)	Hold a little block to move	Put the block in the target scope	59 ± 1	70 ± 2	19%
(13)	Hold a large ball to move	Put the ball in the target scope	64 ± 2	79 ± 2	23%
(14)	Hold a little ball to move	Put the ball in the target scope	59 ± 1	71 ± 2	20%

Category I (Numbers 1–9, tasks without usage of instruments) contains the training items for the first 2 weeks; Category II (Numbers 10–14, tasks with usage of instruments) contains the training items for the latter 2 weeks. *One action per beat, except for those indicated otherwise in the specification. ^#^The average tempo obtained at the baseline; ^§^ the average tempo achieved at the end of the study. bpm, beats per minute.

### Clinical Assessments

The assessments included (I) the Fugl-Meyer Assessment of the affected upper extremity (FMA-UE), (II) the WMFT of the affected upper extremity, (III) the BI, and (IV) the sEMG recordings of the biceps and the triceps on the affected side. The assessments were performed before and after all the treatments. The assessors were blind to patient allocation.

#### Assessments of Upper Extremity Motor Function and ADL

The Fugl-Meyer Assessment (upper extremity) is a scale with high validity to assess motor functions of the upper extremity (Page et al., 2012). In the FMA-UE, 33 items were listed with a total score of 66. Unable to complete a required action or no muscle reflex was scored as 0; partially able to complete the action was scored as 1; adequately complete the action was scored as 2. A higher score indicates better functionality.

The WMFT is used to assess the task-oriented function of the upper extremity. In the WMFT, 15 items were listed with a six-grade scale, from 0, meaning no attempt from the tested arm, to 5, meaning being able to perform the task with a relatively normal movement. A higher score indicates better functionality.

The BI is a commonly used scale to evaluate the ability of ADLs. In the BI, 12 items were listed with a total score of 100. A higher score indicates better functionality.

#### Parameters of sEMG

The sEMG signals were recorded through disposable AgCl electrodes by the 8-channel wireless sEMG system (Myo MUSCLE, Noraxon, USA Inc., Scottsdale, AZ, United States) (Mackala et al., 2013; Kim et al., 2014). The electrodes were placed on the affected biceps and the triceps. The participant sat on an armless chair in a quiet room and rested the affected upper extremity on the body side, as the starting position. The participant was instructed to touch the nose and the most front part of the knee back and forth with the index finger for five times. No audio cue was provided during the assessment. The raw sEMG signal was processed based on RMS (peak %) algorithm. The time window was set at 50 ms. The movement stage of the sEMG was determined based on the simultaneous sEMG recording and real-time video of the joint movement in the sEMG system. The peak normalization was used in this study rather than the maximum voluntary contraction since the paretic limb was tested (Kim et al., 2011). The parameters used in this study were the co-activation interval and the co-contraction index, reflecting the appropriateness of the activation and coordination between the muscles (Thaut et al., 1991; Rosa et al., 2013). The co-activation interval is a ratio of the time of co-activation of the agonist and the antagonist to the total activation time during elbow flexion and extension. The co-contraction index is a ratio of the RMS of the antagonist to the RMS of the agonist. The RMS of the individual muscle groups was also recorded and presented.

### Statistical Analysis

Data analysis was performed by SPSS 22.0 (IBM Corp., Armonk, NY, United States). For the categorical data, chi-square test, or Fisher’s exact test when sample size was below 5, was performed. For the normally distributed continuous data with equal variance, independent *t*-test for comparisons between groups and paired sample *t*-test for comparisons within the group before and after the treat were used. The mix model analysis of covariance (ANOVA) was used to explore time × group interaction. The significance level was set at *p* < 0.05 (2-tailed). G^∗^Power Version 3.1.9.2 (program written by Franz Faul, Universität Kiel; Germany) was used to calculate the sample size and the effect size.

## Results

### Demographics

**FIGURE 1:**
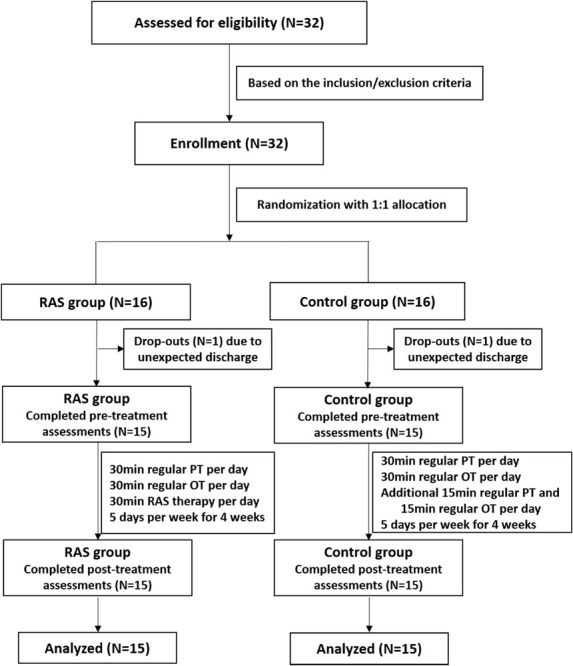
The flow diagram of the study.

Thirty-two patients were initially enrolled in the study, including 16 patients in the RAS group and 16 patients in the control group based on the randomization. One participant in each group was excluded due to unexpected discharge before the pre-treatment assessment. A total of 15 patients in each group completed the study, as shown in Figure 1. Most patients were male, were in their 60s, had ischemic stroke within 6 months, and classified as Brunnstrom Stage IV on enrollment. All patients were right-handed. Primary stroke area was basal ganglia in both groups. No statistical difference was found in the demographics between the two groups (Table 2).

**TABLE 2:** The demographics of the participants.

	**RAS group (*n* = 15)**	**Control group (*n* = 15)**	***P*-value**
Age (years)	66.67 ± 13.59	64.40 ± 13.41	0.821
Gender (male:female)	13:2	10:5	0.195
Type (ischemic:hemorrhagic)	10:5	12:3	0.409
Brunnstrom stage (IV:V:VI)	9:4:2	10:3:2	0.907
Handedness (left:right)	0:15	0:15	1.000
Damage hemisphere (left:right)	7:8	8:7	0.833
Time (months)	5.00 ± 7.55	3.77 ± 9.00	0.137
Intracranial lesion (number,%)			0.707
Frontal temporal lobe	1 (7%)	/	
Frontal lobe	/	1 (7%)	
Corona radiate	1 (7%)	/	
Capsula externa	1 (7%)	/	
Thalamus	2 (13%)	1 (7%)	
Basal ganglia	9 (59%)	8 (52%)	
Brainstem	1 (7%)	3 (20%)	
Paraventricular	/	1 (7%)	
Cerebellum	/	1 (7%)	

**RAS, rhythmic auditory stimulation*.*

Among the Category I tasks, the tempo for most of the items advanced around 50% at the end of the study, except for movements involving shoulder horizontal abduction/adduction and forearm pronation/supination. Among the Category II tasks, the tempo for most of the items advanced around 20% (Table 1).

### Motor Functions of the Hemiparetic Upper Extremity

Motor function assessment, using the FMA-UE, showed improvement after the treatments in both groups, with 20% increase in the RAS group (*p* = 0.000) and 12.5% increase in the control group (*p* = 0.000). There was no statistical difference between groups before the treatments (*p* = 0.542). No statistical difference reached between groups after the treatments (*p* = 0.052). However, the ANOVA analysis revealed significant effect of treatment over time [group × time interaction: *F*(1,28) = 7.717, *p* = 0.010]. The score was higher in the RAS group than that of the control group after the treatments with an effect size of 0.52.

Task-oriented movement, using the WMFT, showed improvement after the treatments in both groups, with 32% increase in the RAS group (*p* = 0.000) and 16% increase in the control group (*p* = 0.000). There was no statistical difference between groups before the treatments (*p* = 0.591). There was statistical difference between groups after the treatments (49.53 ± 10.56 in the RAS group vs. 42.67 ± 10.20 in the control group, *p* = 0.041; effect size, 0.72). The ANOVA analysis also revealed significant effect of treatment over time [group × time interaction: *F*(1,28) = 14.526, *p* = 0.001]. The results are presented in Table 3.

**TABLE 3:** Motor functions and activity of daily living assessments.

	**RAS group**		**Control group**	
	**Pre-treatment**	**Post-treatment**	**Pre-treatment**	**Post-treatment**
**FMA-UE**	50.40 ± 9.97	59.73 ± 6.23*	48.27 ± 8.93	54.07 ± 8.85*
**WMFT**	38.27 ± 8.75	49.53 ± 10.56*^#^	36.60 ± 8.00	42.67 ± 10.20*
**BI**	60.67 ± 10.33	80.33 ± 8.96*^#^	60.33 ± 6.40	69.67 ± 7.19*

**^∗^p < 0.05 between pre- and post-treatment in the same group; ^#^p < 0.05 between groups with the same scale after the treatments. RAS, rhythmic auditory stimulation; FMA-UE, Fugl-Meyer Assessment—Upper extremity; WMFT, Wolf Motor Function Test; BI, Barthel Index*.*

### Activities and Participation

The ADLs, using the BI, showed significant improvement after the treatments in both groups, with 31% increase in the RAS group (*p* = 0.000) and 17% increase in the control group (*p* = 0.000). There was no statistical difference between groups before the treatments (*p* = 0.916). Statistical difference was found between groups after the treatments (80.33 ± 8.96 in the RAS group vs. 69.67 ± 7.19 in the control group, *p* = 0.001; effect size 0.91). The ANOVA analysis also revealed significant effect of treatment over time [group × time interaction: *F*(1,28) = 23.197, *p* = 0.000]. The results are presented in Table 3.

### Surface EMG Features of the Biceps and the Triceps During Elbow Flexion and Extension

#### Co-activation Interval

The results are presented in Table 4. During elbow extension, no statistical difference was found between the two groups before the treatments (*p* = 0.468). Statistical significance was found between groups after the treatments (26.70 ± 13.59 in the RAS group vs. 51.58 ± 21.46 in the control group, *p* = 0.022; effect size, 0.64). There was a 48% reduction in the co-activation interval in the RAS group before and after the treatments (*p* = 0.031), but no significant reduction in the control group (*p* = 0.562). The ANOVA analysis revealed significant effect of treatment over time [group × time interaction: *F*(1,28) = 11.539, *p* = 0.002].

**TABLE 4:** Surface EMG features of the biceps and the triceps during elbow flexion and extension.

	**RAS group**		**Control group**	
	**Pre-treatment**	**Post-treatment**	**Pre-treatment**	**Post-treatment**
**Co-activation interval (%)**				
Elbow extension	51.54 ± 15.62	26.70 ± 13.59*	52.02 ± 21.03	51.58 ± 21.46^#^
Elbow flexion	26.81 ± 18.25	14.80 ± 7.79*	32.23 ± 21.95	28.66 ± 20.82^#^
**RMS during elbow extension**				
Biceps	32.05 ± 11.53	34.87 ± 9.18	32.40 ± 10.83	38.07 ± 11.05
Triceps	36.79 ± 10.32	40.07 ± 10.83	38.87 ± 10.89	38.67 ± 9.88
Co-contraction index (%)	94.27 ± 46.01	92.02 ± 30.46	85.93 ± 29.75	103.67 ± 38.33
**RMS during elbow flexion**				
Biceps	43.15 ± 11.01	44.03 ± 10.07	46.39 ± 6.86	47.00 ± 4.66
Triceps	41.29 ± 14.07	46.33 ± 13.18	46.69 ± 11.56	40.47 ± 13.83
Co-contraction index (%)	101.86 ± 42.53	112.83 ± 49.57	102.28 ± 34.01	86.68 ± 30.24

*p < 0.05 between pre- and post-treatment in the same group; ^#^p < 0.05 between groups with the same movement after the treatments. RAS, rhythmic auditory stimulation; RMS, root mean square. Co-contraction index during elbow extension was calculated as the RMS of the antagonist (biceps)/the RMS of the agonist (triceps); co-contraction index during elbow flexion was calculated as the RMS of the antagonist (triceps)/the RMS of the agonist (biceps).

During elbow flexion, similarly, no statistical difference was found between the two groups before the treatments (*p* = 0.944). Statistical significance was found between groups after the treatments (14.80 ± 7.79 in the RAS group vs. 28.66 ± 20.82 in the control group, *p* = 0.001). There was a 44% reduction in the co-activation interval in the RAS group before and after the treatments (*p* = 0.000), while a 9% reduction in the control group (*p* = 0.930). The ANOVA analysis revealed no significant effect of treatment over time [group × time interaction: *F*(1,28) = 1.091, *p* = 0.305].

#### Co-contraction Index

No statistical difference was found between groups before or after the treatments, or in the individual group before and after the treatments. In the RAS group, during elbow flexion, the co-contraction index increased after the treatments (from 101.86 to 112.83%), while it decreased during elbow extension (from 94.27 to 92.02%). In the control group, an inversed pattern was observed, where during elbow flexion, the co-contraction index decreased after the treatments (from 102.28 to 86.68%), while it increased during elbow extension (from 85.93 to 103.67%). During elbow flexion, the ANOVA analysis revealed no significant effect of treatment over time [group × time interaction: *F*(1,28) = 1.887, *p* = 0.180], or during elbow extension [group × time interaction: *F*(1,28) = 2.001, *p* = 0.168]. The results are presented in Table 4.

#### RMS of the Biceps and the Triceps During Elbow Flexion and Extension

The RMS of the biceps and the triceps during elbow flexion and extension was obtained as an indirect parameter of muscle recruitment. No statistical difference was found before and after the treatments in both groups. The results are presented in Table 4.

## Discussion

The study showed the potential benefits of using RAS as an adjuvant therapy among those who have achieved relatively higher functional levels on the hemiparetic upper extremity, when compared with a control group that only received regular therapies. Our results provided confirmatory evidence on the benefits of clinical application of RAS for post-stroke upper extremity motor restoration and proposed the potential underlying higher increments in the triceps recruitments to the biceps on sEMG.

### Upper Extremity Functional Improvements After RAS Therapy

The participants in the study were required to achieve Brunnstrom IV or above as most of the tasks in our RAS protocol required isolated joint movements. Theoretically, with the decreasing spasticity and the emerging isolated movements, the patients gradually regain the liberty and functional abilities of the limb (Naghdi et al., 2010). However, movement is not only about tone and strength; it is also about preparation, coordination, accuracy, stability, and efficiency, thus a complex and integrated process (Arciniegas et al., 2018). Using RAS in task-oriented exercises may help stimulate rhythm perception in the vestibular system and subsequently relay activations to the cerebella and the premotor area, which eventually transmitted to the internal “beat keeper” pathway, including basal ganglia and supplementary motor area, to assist motor production in good quality (Todd and Lee, 2015). The regimen using RAS reported before showed similar benefits in FMA (Malcolm et al., 2009; Thielman, 2010). In chronic stroke (≥ 4 months), the clinically importance difference (CID) for patients with mild to moderate upper extremity hemiparesis (baseline FMA-UE ≥ 28 and ≤50) was considered to be 4.25–7.25 points (Page et al., 2012), while in the relatively acute stage (on average 1.5 months after stroke onset), with moderate to severe hemiparesis (baseline FMA-UE scores around 14), the CID could be 9–12.4 points (Hiragami et al., 2019). The minimal CID of the WMFT was 3–6 points in chronic stroke patients (≥6 months) (Lin et al., 2009). The minimal CID of the BI was 1.85 points (Hsieh et al., 2007). The patients in the control group showed functional improvements comparable with the above proposed CIDs. The patients in the RAS group had more prominent improvements than those in the control group, suggesting additional clinical benefits of using RAS. The effect size of the FMA-UE was not as high as expected, which may be related to the higher functional baseline and the ceiling effect. The functional improvements were also reflected on the increments of the tempo achieved at the end of the study, which may be an indirect manifestation of the improved efficiency, coordination, and motor priming of the hemiparetic upper extremity. Other researchers combined RAS with bilateral arm training also showed promising results on strength, range of motion and motor performance of the affected arm (Whitall et al., 2000; Cauraugh and Summers, 2005), although there were reports on its ineffectiveness (Richards et al., 2008; Mainka et al., 2018). Moderate to significant discrepancies existed among the regimens in terms of the audio type (metronome/use of music), the duration and frequency of the treatment, the aimed body structures (wrist/elbow/shoulder/fingers), the choice of the rhythm regime (participants’ preference/increase by certain percentage), and the concomitant exercise paradigm (Ghai, 2018), as well as patient selection. The discrepancies make comparisons on the effects difficult. The abovementioned RAS components should be taken into account in the future studies and clinical application. Currently, the duration and frequency of RAS therapy were recommended to be at least 30 min to 1 h per session, more than 3 sessions per week, and more than 10 sessions in total (Ghai, 2018). Based on the results, our RAS regimen is considered feasible for clinical application in the selected population. The detailed RAS regimen and expected advancement of the therapy were also provided for future reference (Table 1).

### Co-contraction Features on sEMG During Elbow Flexion and Extension After RAS Therapy

Spastic co-contraction of the agonist and the antagonist is a common pathological change after stroke (Lin et al., 2009). It is characterized by undesired involuntary muscle activity in the antagonist during voluntary recruitment of the agonist, which makes isolated movements difficult (Baude et al., 2019; Chalard et al., 2020). Our results revealed significant reduction in the co-activation interval following RAS therapy, especially during elbow extension. In a previous study, the sEMG signals of the biceps and triceps were obtained from healthy subjects during elbow bi-articular movements. The co-activation interval during elbow extension and flexion appeared to be around 30–40% and 10–15%, respectively (Thaut et al., 1991). RAS therapy may help modulate tonicity and motor planning (Schaffert et al., 2019), which appeared to bring the agonist/antagonist activation pattern close to normal. The conventional therapy did not show such benefit. The between-group difference after treatment during elbow flexion was not supported by the group–time effect, maybe because of less prominent changes over time, or insufficient sample size, or insufficient treatment duration.

Root mean square was used to reflect the muscle activation signal changes during the elbow bi-articular voluntary tasks. A previous study showed that smoother motor performance was achieved with the restoration of the time-domain reciprocal EMG activities in bi-articular arm muscles (Miyoshi et al., 2010). Based on the formula, a decrease in the co-contraction index was expected to reflect the dominating agonist activation (Kiewiet et al., 2017). In our study, although no statistical significance was found in the RMS of either biceps or triceps, or their respective co-contraction index during elbow flexion or extension, an inversed change was observed in the co-contraction indexes before and after the treatments between groups. A decrease of the co-contraction index in the RAS group was found, compared with an increase in the control group during elbow extension, while an increase in the RAS group was found, compared with a decrease in the control group during elbow flexion. It indicated higher recruitments of the triceps, either as an agonist during elbow extension or as an antagonist during elbow flexion, than that of the biceps after RAS therapy. It may be explained by a greater volitional control of the triceps achieved during elbow extension as the primary agonist and an evidence of improved eccentric contraction of the triceps during the elbow flexion, which might contribute to well-controlled movements. Further studies with larger sample size and longer treatment duration are needed to confirm these findings.

### Limitations

The study was explorative to reveal the potential role of RAS in post-stroke upper extremity motor rehabilitation. The sample size of the study was relatively small but was comparable with previous studies and validated by sample size calculation. Most of the participants reached Brunnstrom IV–VI as it is required to perform the RAS therapy items, which is usually considered relatively high functional level in the recovery process. The distribution of the demographics and the baseline functions was comparable between groups, and the homogeneity of the functional outcomes was acceptable. Therefore, the statistical significance in the functional outcomes between groups may be mainly attributed to the difference in interventions. However, the study did not examine its efficacy on patients with Brunnstrom I–III, which may require a different RAS regimen to match patient’s functional levels. In the study, multiple comparison correction was not performed for sEMG data as false-positive results from chance was less of a concern in the small sample size explorative study. However, it may exist; therefore, it is important to bring this up to the readers’ attention for further interpretation. The relationship between the sEMG and the force generated from the muscles was not examined in the study, as well as the trajectory of the motion. Further studies are needed with a larger sample size to stratify the effects of RAS on different functional levels and to illustrate its effects on different domains of motor functions. Comparison with a group of healthy age- and gender-compatible subjects is warranted to confirm our sEMG findings.

## Conclusion

Using RAS in task-oriented exercises in post-stroke rehabilitation was effective in moderating co-contraction, facilitating task-oriented movements of the hemiparetic upper extremity, and improving ADLs among those who had emerging isolated joint movements. The effects were evident on sEMG possibly by adjusting the balance of recruitments between the agonist and the antagonist.

## Data Availability Statement

The datasets generated for this study are available on request to the corresponding author.

## Ethics Statement

The studies involving human participants were reviewed and approved by Ethics Committee of Huashan Hospital, Fudan University. The patients/participants provided their written informed consent to participate in this study.

## Author Contributions

RT and YZ contributed in study design, patient recruitment, therapy supervision, and data collection. RT and BZ contributed in data analysis, data interpretation, and manuscript preparation. All authors contributed to the article and approved the submitted version.

## Conflict of Interest

The authors declare that the research was conducted in the absence of any commercial or financial relationships that could be construed as a potential conflict of interest.

## Abbreviations

ADL: activities of daily living
BI: Barthel Index
CID: clinical important difference
FMA-UE: Fugl-Meyer Assessment—Upper Extremity
MMSE: Mini-Mental State Examination
RAS: rhythmic auditory stimulation
RMS: root mean square
RS: reticulospinal
sEMG: surface electromyography
WMFT: Wolf Motor Function Test.
